# A randomised controlled test in virtual reality of the effects on paranoid thoughts of virtual humans’ facial animation and expression

**DOI:** 10.1038/s41598-024-67534-4

**Published:** 2024-07-24

**Authors:** Shu Wei, Daniel Freeman, Victoria Harris, Aitor Rovira

**Affiliations:** 1 Department of Psychiatry, University of Oxford, Oxford, UK; 2 Department of Experimental Psychology, University of Oxford, Radcliffe Observatory Quarter, Oxford, OX2 6GG UK; 3 Oxford Health NHS Foundation Trust, Oxford, UK; 4 Nuffield Department of Primary Care Health Sciences, University of Oxford, Oxford, UK

**Keywords:** Psychology, Human behaviour

## Abstract

Virtual reality (VR) is increasingly used in the study and treatment of paranoia. This is based on the finding that people who mistakenly perceive hostile intent from other people also perceive similar threat from virtual characters. However, there has been no study of the programming characteristics of virtual characters that may influence their interpretation. We set out to investigate how the animation and expressions of virtual humans may affect paranoia. In a two-by-two factor, between-groups, randomized design, 122 individuals with elevated paranoia rated their perceptions of virtual humans, set in an eye-tracking enabled VR lift scenario, that varied in facial animation (static or animated) and expression (neutral or positive). Both facial animation (group difference = 102.328 [51.783, 152.872], *p* < 0.001, ${\eta }_{p}^{2}\hspace{0.17em}$= 0.125) and positive expressions (group difference = 53.016 [0.054, 105.979], *p* = 0.049, ${\eta }_{p}^{2}\hspace{0.17em}$= 0.033) led to less triggering of paranoid thoughts about the virtual humans. Facial animation (group difference = 2.442 [− 4.161, − 0.724], *p* = 0.006, ${\eta }_{p}^{2}\hspace{0.17em}$= 0.063) but not positive expressions (group difference = 0.344 [− 1.429, 2.110], *p* = 0.681, ${\eta }_{p}^{2}\hspace{0.17em}$= 0.001) significantly increased the likelihood of neutral thoughts about the characters. Our study shows that the detailed programming of virtual humans can impact the occurrence of paranoid thoughts in VR. The programming of virtual humans needs careful consideration depending on the purpose of their use.

## Introduction

Paranoia—perceiving hostile intent where there is none—is prevalent in the general population. Many individuals occasionally experience paranoid thoughts, while a smaller number of people frequently experience paranoid thoughts^1^. A recent survey of a representative group of ten thousand UK adults indicated that approximately one in six people wanted help to be more trusting of other people^2^. Virtual reality (VR) has been used to both study^3–7^ and treat^8–11^ paranoia. Freeman et al.^6^ pioneered the use of VR to assess and understand paranoia by examining people's appraisals of neutral virtual humans. The insight was that if the characters are neutral but hostile intent is perceived, then this is clear evidence of paranoid thinking. Studies have shown that higher levels of paranoia in daily life are associated with experiencing higher levels of paranoia about virtual characters^12^. Qualitative findings indicate that close observations of the virtual humans may contribute to the occurrence of paranoid interpretations. As a participant in one study described: “I was just looking around, looking at people, just observing them…”^13^. This paper reports, for the first time, on the detailed characteristics of virtual humans that may affect their appraisal by people vulnerable to paranoia.

Previous VR studies outside of the topic of paranoia have shown that facial expressions and animations of virtual humans can significantly influence people’s behavioural and psychological responses. For example, Geraets et al.^14^ suggested that facial emotion cues are beneficial for non-clinical populations to accurately identify the emotions of virtual characters, with the recognition accuracy in VR comparable to that in photographs and videos. Additionally, it was observed that participants tended to focus more on the eye and nose areas when interpreting the emotions of the virtual humans. Bönsch et al.^15^ looked at how the emotional expressions of virtual humans affect personal space preference, by examining responses to approaches by virtual men exhibiting happy, angry, or neutral expressions on static faces. The study showed that participants maintained larger distances from a virtual man with an angry face compared to a happy or neutral face. Moreover, Kimmel et al.^16^ found that integrating facial animations, such as mouth and eye movements, not only enhanced the social presence in the scenario felt by participants but also made participants feel that the virtual humans had a better understanding of their emotions and attitudes.

Notably, recent investigations have shown the significant role of virtual human faces in shaping the perception of trust in VR^17–19^. Luo et al.^18^ suggested that, compared to neutral or negative facial expressions, positive facial expressions promoted trust and willingness to cooperate in a VR game. Choudhary et al.^17^ explored the impact of conflicting facial and vocal emotional expressions. They found that virtual humans with happy faces were perceived as happier and more trustworthy than virtual humans with unhappy faces. Although appraisals of trust became less predictable for mismatched expressions (e.g., a happy face with unhappy voice), facial expressions had a stronger impact than vocal tone. Wei et al.^19^ found that adding positive facial expressions to a virtual coach in a VR phobia treatment significantly improved user connection with that coach. Furthermore, animated faces of VR characters have been found to be perceived as more natural and believable than static faces in VR social experiences^16,20^.

Despite the evidence of the impact of virtual human characteristics on user perceptions, the influence of the detailed programming of virtual human faces on paranoia remains untested. In this study, we focused on two key elements: facial animation and facial expression. Previous research has indicated that facial animation can make virtual humans appear more empathetic and less strange^21^, while positive facial expressions can promote trust^18^. Therefore, our primary hypothesis was that using facial animation or positive facial expression would reduce the likelihood of paranoia appraisals. VR eye-tracking enables capture of objective data of where a person is looking^14,22^. Hence, we also examined eye tracking data to provide information about how individuals vulnerable to paranoia may pay visual attention to virtual characters.

## Methods

### Experimental design

**Figure 1:**
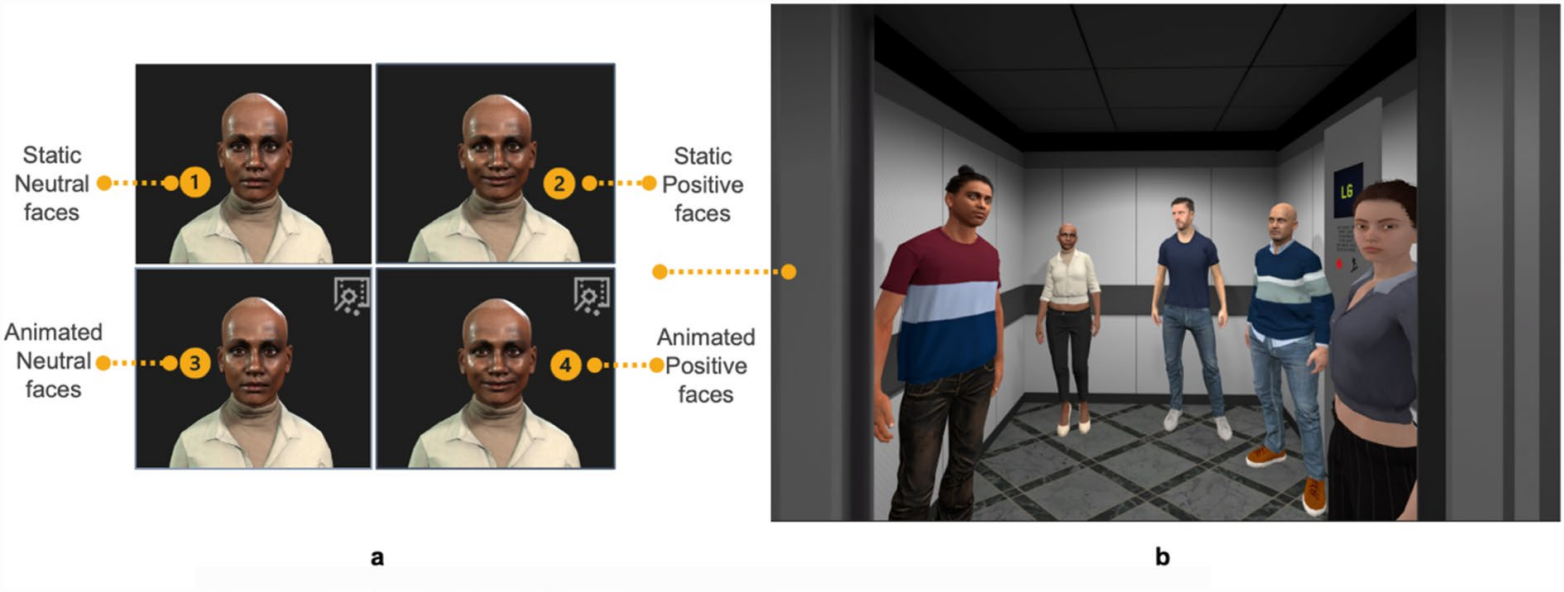
(**a**) Factorial design with virtual human faces varied in animation (static or animated) and expressions (neutral or positive) (**b**). VR lift environment: view of the lift when the door opened with five virtual humans standing inside.

We used a two-by-two factorial, between-groups, randomised design to examine two aspects of facial programming: animation (static or animated) and expression (neutral or positive). Participants were randomised to one of the four experimental conditions (see Fig. 1a): all virtual humans having (1) static neutral (2) animated neutral (3) static positive or (4) animated positive faces. In all experimental conditions, the virtual humans had the same body animation. The study was conducted single-blind: participants were unaware of the study hypotheses and that they were randomized into one of the different versions. The randomization was conducted by an independent researcher using *Research Randomizer*^23^.

### Apparatus and VR scenario

We used a Windows 10 computer (Intel i7-8700K, Nvidia GeForce GTX 1080Ti, 32 GB RAM) to run the VR scenario and render it on a *Meta Quest Pro* (Meta, 2022) via Meta Air Link. The headset has a resolution of 1832 * 1920 pixels per eye and a field of view of 106° (horizontal) × 96° (vertical). The refresh rate was set at 90 Hz refresh rate.

The VR software was developed in Unity 2021.3.15 with Oculus’ Movement SDK V1.3.2, presenting a virtual lift scenario. Participants began in a hallway waiting for the lift to arrive. The lift door opened automatically upon arrival, and participants were instructed to take the lift to the “sky lounge”. Inside the lift were five virtual humans (three men and two women of different ages and ethnicities), as shown in Fig. 1b. The ride lasted three minutes, and the VR scenario concluded when the lift reached the “sky lounge”. A video of the VR experience is provided in the supplementary materials.

We implemented the facial animations by combining motion capture technologies and blend shape animation. Starting with motion-captured facial movements to establish a foundational animation, we then applied blend shapes for smoother transitions between expressions. The animations were recorded and refined using *Iclone7* with the *LiveFace* plugin (https://mocap.reallusion.com/iclone-motion-live-mocap/iphone-live-face.html). We also programmed the characters to occasionally look at the participants and make eye contact with them.

We used the integrated eye tracker in the *Meta Quest Pro* to monitor eye gaze behaviour in VR. We pre-defined a set of regions of interest (ROIs) in the VR environment to record the eye gazes directed at them. The ROIs included the faces of all five virtual humans and other specific areas where participants might look to avoid eye contact—the floor, the screen in the lift displaying the current floor, and the exit door. The software was adjusted to minimize false negatives when detecting eye fixation to any of the elements on the ROI list. We accounted for the accuracy reported of the Meta Quest Pro’s eye tracker (accuracy: 1.652°; SD precision: 1.652°)^24^.

### Participants and recruitment

Participants were recruited via social media advertisements in Oxfordshire, United Kingdom. We screened for individuals vulnerable to paranoia using *The Revised Green *et al*., Paranoid Thoughts Scale (R-GPTS)*^25^, with a Part B score greater than 5. This cut-off score captures elevated or higher levels of persecutory ideation. Exclusion criteria were individuals (a) under 18 years old, (b) reported photosensitive epilepsy in the past or a significant visual, hearing, or mobility impairment that would prevent them from using VR, or (c) currently under medication that could induce motion sicknesses. Participants requiring correction-to-normal lenses were requested to use contact lenses to avoid any potential discomfort wearing the VR headset and to preserve the quality of the data recorded with the eye tracker.

Ethical approval was received from the University of Oxford Medical Sciences Interdivisional Research Ethics Committee (R85111/RE001). The study was performed in accordance with relevant guidelines and regulations. Written informed consent was obtained from all participants.

1581 individuals completed the screening questionnaire, 296 were eligible (i.e. adults with elevated paranoia). 122 participants (female = 70, male = 52) attended the VR session (we had booked an extra two participants over the target sample size to account for potential cancellations).The average age of the participants was 36.2 years (SD = 14.8, range: 18, 76). The mean R-GPTS Part B score was 13.30 (SD = 7.70, range: 6, 37). The average previous experience of VR rated on a 5-point-scale (where 1 indicates “never tried VR” and 5 indicates “very experienced”) was 2.00 (SD = 1.13). Table 1 provides a summary of the participants’ demographic information and study-relevant data.

**Table 1:** Participant information per group.

	Static neutral (n = 31)	Static positive (n = 30)	Animated neutral (n = 30)	Animated positive (n = 31)
Mean age in years (SD)	36.2 (16.3)	36.1 (13.6)	36.8 (13.9)	35.9 (15.9)
Gender				
Male (%)	12 (38.7%)	12 (40.0%)	15 (50.0%)	13 (41.9%)
Female (%)	19 (61.3%)	18 (60.0%)	15 (50.0%)	18 (58.1%)
Ethnicity				
White	25	18	22	25
Black/African American	0	1	0	0
Asian	6	7	6	6
Others	0	4	2	0
R-GPTS Part B (i.e. baseline paranoia score) (SD)	13.35 (7.71)	13.07 (7.31)	14.00 (8.18)	12.81 (7.89)
Previous experience of VR (SD)	2.00 (0.93)	2.07 (1.28)	2.40 (1.19)	2.19 (1.17)

### Experimental procedures

Each participant was invited to the university for a single session at our VR lab. The researcher provided an overview of the study procedure and informed participants that they would try out a VR social experience, during which eye gaze direction data would be collected (no pictures or videos of their eyes would be recorded). Participants gave written informed consent to participate. The researcher then helped the participants fit the VR headset and guided them through the eye tracker calibration process. The researcher then selected the parameters according to each participant’s experimental condition group and they experienced the VR lift ride. Once the VR scenario ended, participants took the VR headset off and completed the paranoid thoughts visual analogue scales^26^ and State Social Paranoia Scale^27^. Finally, they were debriefed about the full purpose of the study. The session lasted approximately 45 min, and participants were reimbursed for their time.

### Measures

*Baseline paranoia* During screening participants completed *The Revised Green *et al*., Paranoid Thoughts Scale (R-GPTS)*^25^. We used Part B to assess ideas of persecution. There are 10 items such as “I was sure someone wanted to hurt me” and “People have been hostile towards me on purpose”. All items are scored from 0 (Not at all) to 4 (Totally), with total scores ranging from 0 to 40 (Cronbach’s α = 0.86 in the current study, N = 122). Higher scores reflect higher levels of paranoia.

*Paranoid thoughts visual analogue scales (VAS)*^26^ This was the *primary* paranoia outcome measure, assessing participants’ appraisals of the virtual humans in the lift scenario. After the VR experience, participants rated 6 visual analogue scales concerning the VR humans (“Right now I feel suspicious of the people in the lift”, “Right now I feel that people in the lift wanted to harm me”, “Right now I feel like the people in the lift wanted to upset me”, “Right now I feel like the people in the lift were against me”, “Right now I am thinking that the people in the lift were trying to persecute me” and “Right now I feel like the people in the lift were hostile towards me”). Participants marked each item on a standard 10 cm visual analogue scale on paper from 0 (not at all anxious) to 100 (extremely), with total scores ranging from 0 to 600 (Cronbach’s α = 0.935 in the current study, N = 122). Higher scores indicate higher levels of paranoia about the virtual humans.

*State social paranoia scale (SSPS)*^27^ This provided a further assessment of paranoid thoughts about the virtual human and also neutral and positive appraisals. In the scale, each item is scored from 1 (Do not agree) to 5 (Totally agree). There are 10 items measuring paranoid thoughts (SSPSPersecutory) (range: 10, 50, Cronbach’s α = 0.950 in the current study, N = 122), and 5 items each measuring neutral views (SSPSNeutral) (range: 5, 25, Cronbach’s α = 0.825) and positive views (SSPSPositive) (range: 5, 25, Cronbach’s α = 0.736) of the people in the VR social situation. Higher SSPS scores on each subscale indicate greater levels of persecutory or neutral or positive thinking.

*Eye gaze data* We recorded the raw eye tracker output and the detected eye gazes on ROIs (i.e. the start and end times for each gaze on an ROI object). Data were first screened for missing records by examining gaps in the raw data output file. We excluded the data from a participant if more than 15% of the data were missing, following the suggested practices in Holmqvist et al. and Schuetz & Fiehler^28,29^. We processed the data to calculate the duration for each eye gaze event, and identified fixations using a time threshold of 0.275 seconds^30^. We then aggregated these fixations for each ROI object per participant. The following variables were calculated for analysis:

- *Visual Attention to Virtual Humans*: total fixation time on virtual humans as the percentage of total fixation time on all ROI objects.
- *Visual Attention to Exit/Floor/Lift Screen (displays the current floor)*: total fixation time on lift exit door/floor/screen as the percentage of total fixation time on all ROI objects.
- *First Fixation Target:* The object participants looked at on the first fixation after entering the lift.

### Statistical methods

We used two-way ANCOVA models, examining the effects on participant views of the virtual humans of facial animation and positive facial expression while controlling for baseline paranoia. We first checked the data against the assumptions of the ANCOVA model, using Levene’s test for homogeneity of variance and Shapiro–Wilk test of normality. We also performed a log transformation on heavily skewed data (visual attention to the floor and screen) before analysis. Details of the assumption checking results are included in the supplementary materials. A similar approach was taken to analysing the eye gaze data.

All significance tests were made at the α = 0.05 level, and we calculated the partial eta-squared (${\eta }_{p}^{2}$) to measure the effect sizes. Tukey’s honest significant difference test (Tukey's HSD) was used for multiple pairwise comparisons with the adjusted *p* value. We report the results as mean group differences and 95% confidence interval (95% CI). Additionally, we conducted contrast tests in cases where a significant interaction was detected, assessing the impact of each factor at different levels of another factor, with estimates reported alongside their 95% confidence interval. Data cleaning and processing was performed using *Python*’s *Pandas* and *NumPy* libraries^31,32^. The statistical analysis was done in *R*.

To determine the target sample size for our experimental design, we aimed to detect a medium effect size of partial eta-squared = 0.06 and conventional values of power = 0.80 and α = 0.05 for a between-factors ANOVA using G * power 3.124. Thus, a total of 120 participants (30 per condition) would be required.

## Results

**Figure 2:**
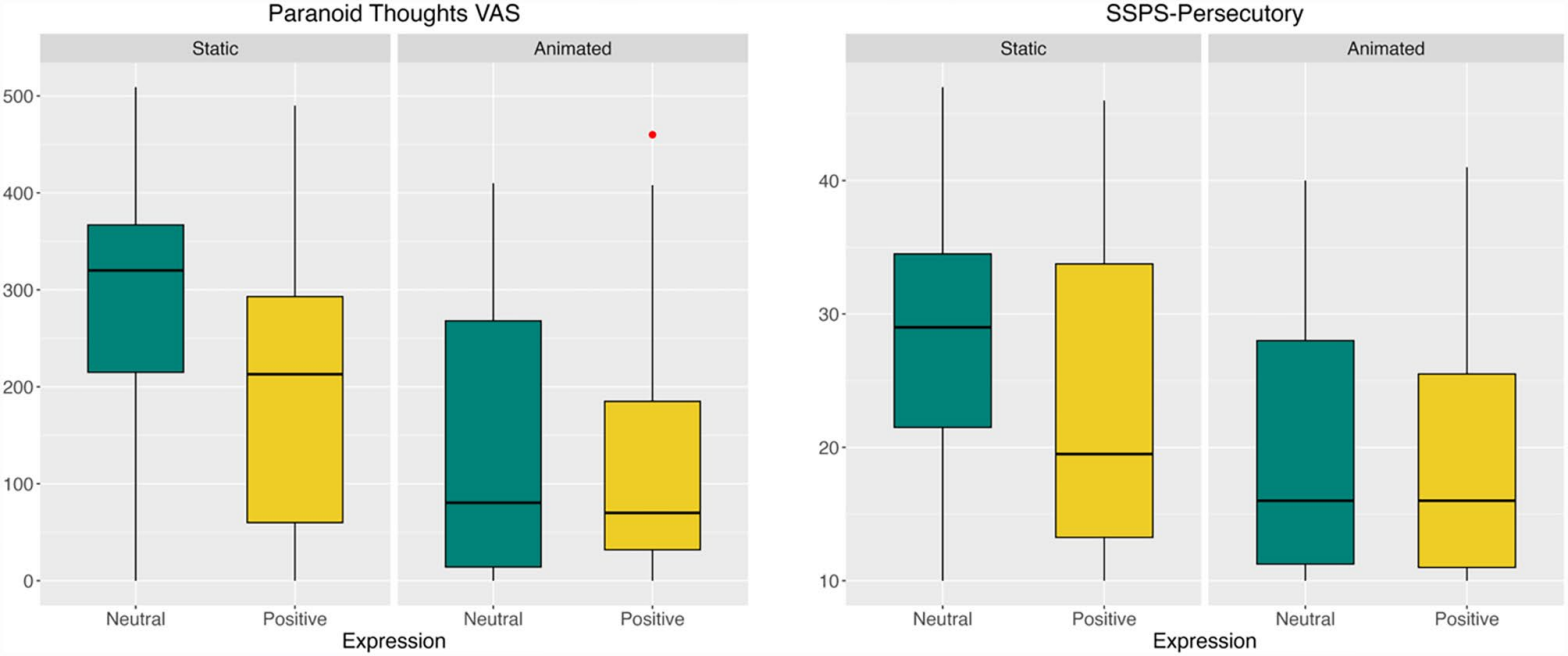
Box plots of the VAS *Paranoia* and *SSPS-Persecutory.* Red point indicates the outlier point.

Table 2 summarises scores for the paranoid thoughts VAS and the three SSPS subscales by randomised group. There were no missing data in these measures. Both the paranoid thoughts VAS and SSPS persecutory were used to assess participants’ paranoid ideation and were positively correlated (Spearman r = 0.82, *p* < 0.001). Figure 2 shows summary scores for these two measures in the randomised groups.

**Table 2:** Descriptive statistics of the appraisals of the virtual humans by randomisation group.

	Static neutral (n = 31)	Static positive (n = 30)	Animated neutral (n = 30)	Animated positive (n = 31)
VAS Paranoia mean (SD)	285.00 (126.15)	195.33 (146.47)	145.20 (141.88)	132.16 (138.66)
SSPSPersecutory mean (SD)	28.71 (10.86)	20.20 (10.60)	23.03 (10.89)	19.52 (9.80)
SSPSNeutral mean (SD)	11.03 (4.56)	11.87 (4.26)	14.70 (5.08)	13.10 (5.19)
SSPSPositive mean (SD)	11.03 (3.69)	10.87 (4.36)	11.27 (4.17)	14.23 (4.28)

### Paranoid thoughts visual analogue scale (VAS paranoia)

A two-way ANCOVA model was used to assess the impact of facial animation and expressions while controlling for baseline paranoia. Simple main effects analysis showed that facial animation (group difference = 102.328, 95% CI = [51.783, 152.872], F(1, 117) = 17.071, *p* < 0.001, ${\eta }_{p}^{2}$ = 0.125) and positive expression (group difference = 53.016, 95% CI = [0.054, 105.979], F(1, 117) = 3.938, *p* = 0.049, ${\eta }_{p}^{2}$ = 0.033) led to less paranoid thinking about the virtual humans. There was no significant interaction between animation and positive expression (F(1, 117) = 2.519, *p* = 0.115, ${\eta }_{p}^{2}$ = 0.021). The effect of baseline paranoia was not significant (F(1, 117) = 2.678, *p* = 0.104, ${\eta }_{p}^{2}$ = 0.022). Tukey’s HSD test for multiple comparisons showed there was a significant difference between the static neutral and animated neutral group (*p*-adj < 0.001) and between the static neutral and animated positive group (*p*-adj < 0.001).

### SSPS-persecutory thoughts

Simple main effects analysis showed that facial animation (group difference = 6.066, 95% CI = [2.247, 9.885], F(1, 117) = 10.464, *p* = 0.002, ${\eta }_{p}^{2}$ = 0.081) but not positive expressions (group difference = 3.279, 95% CI = [− 0.650, 7.207], F(1, 117) = 2.534, *p* = 0.114, ${\eta }_{p}^{2}$ = 0.021) led to significantly lower levels of paranoia. There was no significant interaction between animation and positive expressions (F(1, 117) = 1.897, *p* = 0.171, ${\eta }_{p}^{2}$ = 0.016). The effect of baseline paranoia was not significant (F(1, 117) = 3.624, *p* = 0.059, ${\eta }_{p}^{2}$ = 0.030). Tukey’s HSD test for multiple comparisons showed a statistically significant difference between the static neutral group and animated neutral group (*p*-adj = 0.008), and between the static neutral and animated positive group (*p*-adj = 0.005).

### SSPS-neutral thoughts

Simple main effects analysis showed that facial animation (group difference = 2.442, 95% CI = [− 4.161, − 0.724], F(1, 117) = 7.843, *p* = 0.006, ${\eta }_{p}^{2}$ = 0.063) but not positive expressions (group difference = 0.344, 95% CI = [− 1.429, 2.110], F(1, 117) = 0.17, *p* = 0.681, ${\eta }_{p}^{2}$ = 0.001) led to a more neutral interpretation of the virtual humans. There was no significant interaction between animation and positive expressions (F(1, 117) = 1.914, *p* = 0.169, ${\eta }_{p}^{2}$ = 0.016). The effect of baseline paranoia was not significant (F(1, 117) = 0.442, *p* = 0.508, ${\eta }_{p}^{2}$ = 0.002). Tukey’s HSD test for multiple comparisons showed there was a statistically significant difference between the static neutral group and animated neutral group (*p*-adj = 0.019).

### SSPS-positive thoughts

There was a significant interaction between animation and positive expressions (F(1, 117) = 4.297, *p* = 0.040, ${\eta }_{p}^{2}$ = 0.035). Facial animation led to more positive thoughts when the expressions were positive (estimate = 3.358 [1.250, 5.460], SE = 1.060, *p* = 0.002), but not when expressions were neutral (estimate = 0.236 [− 1.870, 2.340], SE = 1.060, *p* = 0.825). Positive expressions led to more positive thoughts when the faces were animated (estimate = 2.956 [0.849, 5.060], SE = 1.060, *p* = 0.006), but not when faces were static (estimate = − 0.166 [− 2.271, 1.940], SE = 1.060, *p* = 0.876). Tukey’s HSD test for multiple comparisons showed that there was a statistically significant difference between the static neutral and animated positive group (*p*-adj = 0.016), the static positive and animated positive group (*p*-adj = 0.011), and between the animated neutral and animated positive group (*p*-adj = 0.032).

### Paranoid thinking in VR and baseline paranoia

The correlations between paranoid thinking in VR and baseline paranoia were examined in the different groups through spearman correlation (see Table 3). A moderate positive correlation was found between paranoid thinking in VR and baseline paranoia within the animated neutral group.

**Table 3:** Correlation between VAS paranoia and baseline paranoia.

	Spearman correlation	*p* value
Static neutral (n = 31)	− 0.110	0.554
Static positive (n = 30)	0.000	0.999
Animated neutral (n = 30)	0.385	0.036
Animated positive (n = 31)	0.194	0.296

### Visual attention

**Figure 3:**
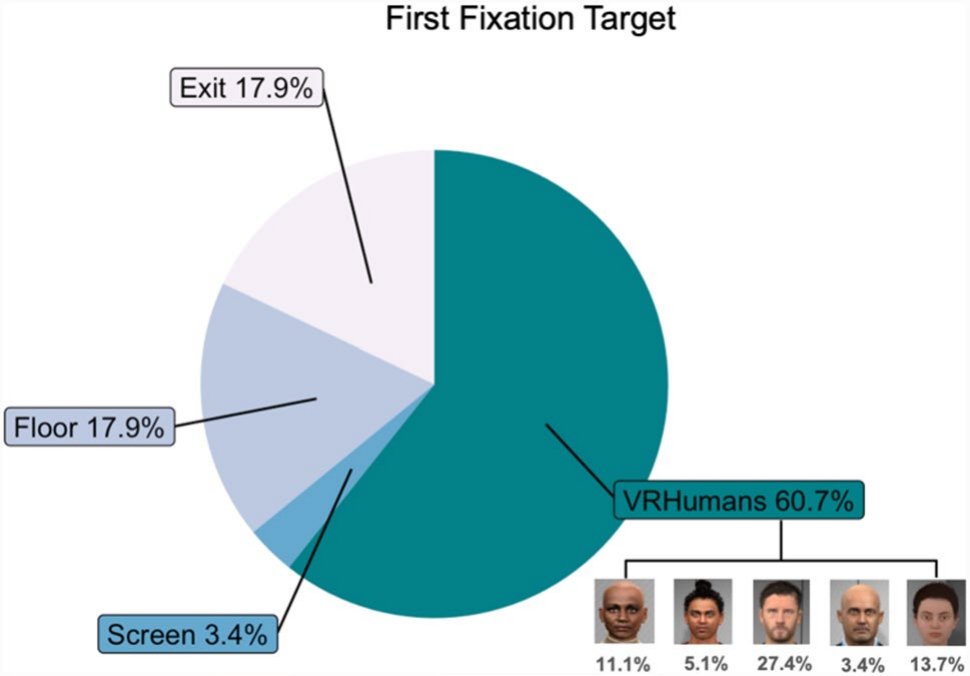
Distribution of the target of first fixation.

Five datasets from the eye-tracking analysis were excluded as more than 15% of the raw data were missing due to technical issues. Analysis of the remaining 117 participants during the lift ride showed an average total fixation duration of 112.59 s (SD = 28.42) and an average of 1.86 s (SD = 1.84) per fixation. Participants spent 31.95% of the fixation time on the virtual humans (SD = 24.68%). The average duration for these fixations was 5.87 s (SD = 5.84). The most common initial fixation targets were the male virtual human directly facing the lift entrance (27.4%), the exit (17.9%), and the floor (17.9%) (see Fig. 3). Descriptive statistics for the visual attention allocation are shown in Table 4.

**Table 4:** Visual attention to virtual humans, lift exit, lift floor, and lift screen.

Visual attention allocation (%)	Static neutral (n = 30)	Static positive (n = 28)	Animated neutral (n = 30)	Animated positive (n = 29)
VR humans (SD)	38.8 (20.6)	21.9 (19.7)	32.6 (27.3)	33.8 (27.9)
Exit (SD)	23.8 (21.2)	41.8 (30.8)	41.9 (36.6)	26.7 (29.3)
Floor (SD)	9.7 (18.3)	8.1 (15.5)	7.4 (14.5)	9.0 (11.6)
Screen (SD)	25.7 (19.7)	26.5 (23.4)	16.8 (16.1)	29.1 (26.0)

*Visual attention to the virtual humans* was tested using a two-way ANCOVA model controlling for baseline paranoia. There was a marginally non-significant interaction between animation and positive expressions (F(1, 112) = 3.591, *p* = 0.061, ${\eta }_{p}^{2}$ = 0.031), suggesting a trend where animation and positive expression might jointly influence the amount of visual attention allocated to the virtual humans. Specifically, positive expressions led to a lower amount of visual attention on virtual humans when faces were static (estimate = − 0.169 [− 0.294, − 0.043], SE = 0.064, *p* = 0.010), but not when faces were animated (estimate = 0.001 [− 0.124, 0.127], SE = 0.063, *p* = 0.983). Facial animation did not affect visual attention to virtual humans either when the facial expressions were neutral (estimate = − 0.060 [− 0.184, 0.064], SE = 0.062, *p* = 0.338) or positive (estimate = 0.110 [− 0.017, 0.238], SE = 0.064, *p* = 0.090). Tukey’s HSD test indicated a significant difference between the static neutral and static positive group (*p*-adj = 0.044).

*Visual attention to the environment* The same two-way ANCOVA model was used to examine the extent of visual attention on the lift exit door, floor, and screen separately. For the exit, there was a significant interaction between animation and positive expressions (F(1, 112) = 8.026, *p* = 0.005, ${\eta }_{p}^{2}$ = 0.067). Facial animation led to a higher amount of visual attention on the exit when the expressions were neutral (estimate = 0.176 [0.027, 0.325], SE = 0.075, *p* = 0.021), but not when expressions were positive (estimate = − 0.131 [− 0.285, 0.023], SE = 0.078, *p* = 0.095). Positive expressions led to a higher amount of visual attention on the exit when faces were static (estimate = 0.182 [0.030, 0.334], SE = 0.077, *p* = 0.020), but not when faces were animated (estimate = − 0.125 [− 0.277, 0.027], SE = 0.077, *p* = 0.105). Tukey’s HSD test indicated no statistically significant differences in pairwise comparisons.

The analysis was performed on log-transformed data for visual attention to the floor and the lift screen. For the floor, there was no significant interaction between animation and positive expressions (F(1, 112) = 0.306, *p* = 0.581, ${\eta }_{p}^{2}$ = 0.003) and there were no main effects from animation (F(1, 112) = 0.025, *p* = 0.874, ${\eta }_{p}^{2}$ < 0.001) or positive expression (F(1, 112) = 0.011, *p* = 0.916, ${\eta }_{p}^{2}$ < 0.001). The effect of baseline mistrust was not significant (F(1, 112) = 2.493, *p* = 0.117, ${\eta }_{p}^{2}$ = 0.020). For the lift screen, there was no significant interaction between animation and positive expressions (F(1, 112) = 1.895, *p* = 0.171, ${\eta }_{p}^{2}$ = 0.017) and there were no main effects from animation (F(1, 112) = 0.846, *p* = 0.360, ${\eta }_{p}^{2}$ = 0.009) or positive expression (F(1, 112) = 2.496, *p* = 0.117, ${\eta }_{p}^{2}$ = 0.019). The effect of baseline mistrust was not significant (F(1, 112) = 1.250, *p* = 0.266, ${\eta }_{p}^{2}$ = 0.009).

*Correlation between visual attention and paranoia* We computed Spearman correlations between visual attention and paranoia using all the retained eye-tracking data across different condition groups (N = 117, see Table 5). There was a positive correlation between the amount of visual attention to the virtual humans and the severity of paranoid thoughts in VR (VAS: r = 0.19, *p* = 0.040) and a positive correlation between the amount of visual attention to the lift exit and baseline paranoia (Baseline Paranoia: r = 0.21, *p* = 0.023). There were no significant correlations between the paranoid thoughts in VR/ baseline paranoia and the visual attention to other ROIs.

**Table 5:** Correlation between visual attention and paranoia measures (N = 117).

Visual attention allocation (%)	Correlation with VAS paranoia		Correlation with baseline paranoia	
	Spearman r	*p* value	Spearman r	*p* value
Virtual humans	0.190	0.040	− 0.046	0.621
Exit	− 0.152	0.101	0.210	0.023
Floor	0.117	0.208	− 0.101	0.277
Screen	− 0.040	0.668	− 0.079	0.398

## Discussion

Our study presented the first test of whether the detailed programming of virtual humans’ facial features affects paranoid interpretations. The primary results supported the hypothesis that facial animation and positive facial expressions of virtual humans both reduce the likelihood of paranoid appraisals. In our study, facial animation and positive expression each independently led to people vulnerable to paranoia perceiving the virtual characters as less hostile. In contrast, paranoid thoughts were more likely to occur when faces were static or the expression was neutral. The sizes of the effects were moderate to large. Facial animation fostered more neutral perceptions of virtual humans too. An examination of the correlation between paranoid thinking in VR and baseline paranoia in each condition indicated that the animated neutral characters produced the strongest association between paranoia in day-to-day life and paranoia in VR. This means that animated neutral characters in VR may provide the most effective assessment test for paranoia. Overall, the study highlights the importance of considering how faces in VR are programmed when assessing or treating paranoia.

The findings align with prior research showing that facial-animated characters appear more natural and believable^20^, and people are better at recognising emotions from them^33^. Similarly, the addition of positive emotion led to the perception of less negative intention or attributes from the virtual characters regardless of whether their faces were animated^14,19^. It was notable that the effects of these two features were independent of people’s baseline paranoia, and in this study, animation had a more substantial effect (accounting for 13% of the variance in paranoia) compared to facial expressions (accounting for 3% of the variance). This might be attributed to our implementation of positive expressions as friendly faces with gentle, subtle smiles, to fit the neutral VR context. Such nuanced emotional expression typically requires accurate delivery with dynamic movement^34^; the absence of animations may lead to the “frozen face” effect, where a static human face appears less flattering than one with motion^35^. Additionally, the lack of dynamic information in the virtual human faces could render their expressions more ambiguous, leading individuals with elevated paranoia to interpret this ambiguity negatively and perceive the virtual humans as potentially hostile^36,37^.

The programming of virtual human faces also influenced individuals’ neutral and positive perceptions. According to Krumhuber et al.^38^, dynamic information (e.g. animation) enhances emotion recognition, particularly when facial expressions are subtle or convey a neutral emotion. Consistent with this, animation led to a more neutral interpretation of the characters, and the animated neutral faces were rated as the most neutral. Interestingly, regarding positive thoughts about the characters, an interaction effect suggested that animation was critical for positive expressions to lead to stronger positive interpretations, while the static positive faces scored the lowest. This reduced likelihood of eliciting positive thoughts from static positive faces likely stems from the mismatch between expressed emotions and the absence of movement, making the characters appear less lively and emotionally inconsistent.

Examining visual attention to virtual humans, we found that positive expressions led to less visual attention when virtual human faces were static. The discrepancy between positive facial expressions and the absence of animation could lead to the characters being perceived as anomalous. Particularly, lack of eye movements, such as eye blinking or gaze, could cause smiles to seem eerie or ungenuine^39,40^. Exploring the link between visual attention and paranoia, we found a positive relationship between attention to virtual humans and paranoid thoughts in VR. This might imply that closely observing virtual humans could foster the development of paranoid ideations^13^, or that individuals with heightened paranoia are more inclined to concentrate on these characters. In addition, there was also a positive relationship between people’s focus on the lift exit and their levels of day-to-day paranoia. This behaviour aligns with the use of safety-seeking strategies in response to persecutory thoughts^41^, verifying that VR elicits reactions similar to those in the real world. Such behaviour also coincides with the patterns found in social anxiety studies, where individuals often avoid eye contact with virtual humans and shift their attention to other areas of the virtual scene under distress^42,43^. Although the complex relationships between visual attention, character animation, and people’s mental health states requires further exploration, the study of eye gaze behaviour might provide additional information to help understand paranoia.

There are several limitations to the study. First, our choice of the VR lift scenario limited participants to a close distance (less than 2 m) from the virtual humans, which may affect the generalizability of the results to scenarios involving greater social distances. A comparison of being in a lift to walking into a room would be of clear interest. Interpersonal distance in VR can affect people’s emotional and behavioural responses^44^. Moreover, the neutral context of strangers in a lift ride may not produce the same results in scenarios with other social interactions. Second, we focused on two features—animation and positive expressions—but other characteristics such as eye gaze behaviour patterns and facial mimicry could also be important^45,46^. Third, we did not consider demographic (e.g. age, gender, ethnicity) similarities or differences between participants and the characters, nor their spatial arrangement. Fourth, our visual attention analysis focused only on spatial allocation and fixation-related metrics, excluding other relevant measures like saccades and gaze angles. Current technology provides limited capability to study whether participants were looking at someone from the corner of their eyes. Additionally, the resolution of the eye tracker was insufficient for a detailed analysis of which facial parts participants focused on (e.g. eyes or mouth). As prior research has shown that people direct their attention to different parts of virtual faces depending on the displayed emotions^14^, further investigation could provide more comprehensive insights into participant behaviours.

Our findings provide evidence that character animations alter people’s perceptions and experiences in VR. This may, for example, affect VR experiences focused on the understanding and treatment of paranoia. Therefore, careful consideration of character design and animation is likely to be important in developing future VR mental health applications.

### Supplementary Information

Supplementary Tables.

Supplementary Video 1.

## Data Availability

Deidentified data are available from the corresponding authors on reasonable request and contract with the university.
